# Fracture-dislocation at C6-C7 level with Quadriplegia after Traditional Massage in a Patient with Ankylosing Spondylitis: A Case Report

**DOI:** 10.5704/MOJ.1707.013

**Published:** 2017-07

**Authors:** KAK Abilash, QMQ Mohd, ZAH Ahmad, Basir Towil

**Affiliations:** Department of Orthopaedics, Hospital Shah Alam, Shah Alam, Malaysia

**Keywords:** ankylosing spondylosis, cervical spine, fracture-dislocation, massage

## Abstract

Ankylosing spinal disorders (ASD) tend to result in fractures and/or dislocations after minor trauma because of the altered biomechanical properties. The relative risk of traumatic vertebral fractures in patients with ankylosing spondylitis has been estimated as three times higher than in the general population. These spine traumas, which are located at cervical level in 81% of patients with ankylosing spondylitis, are complicated by neurological lesions in 65% of patients, due to the high inherent instability of these fractures. Traditional massage is an ancient practice in many parts of Asia. It has many benefits that are currently recognized world-wide. However, it can be dangerous and even lethal if practised without adequate knowledge and skill. We report a case of C6-C7 fracture-dislocation with complete neurology and neurogenic shock in a middle aged man with undiagnosed ankylosing spondylitis.

## Introduction

Ankylosing Spondylitis (AS) is a seronegative spondyloarthropathy that primarily involves the vertebral column and the sacroiliac joints. It is a chronic disease that typically starts before the age of 30 and has a slow but steady progression. The disease has a characteristic caudal to rostral progression and overtime alters the strength and biomechanical properties of the spine through extensive remodelling involving ligamentous ossifications, vertebral joint fusion, osteoporosis and kyphosis^1^. These changes lead to an increase in the segmental rigidity of the spine due to calcifications of soft tissue that predisposes to a risk of pathological fractures affecting the spinal columns, hence resulting in spinal cord injury^2^. Typically, spinal fractures occur mostly in patients with advanced age and therefore have inherently poorer outcome. Cervical spine fractures occur three times more frequently in patients with AS^3^.

The diagnosis of AS is based on clinical and radiographic features. The modified New York Criteria has a high sensitivity and specificity; however, they may not be helpful for early diagnosis of AS and the involvement of the sacroiliac joint remains the sine qua non for definite diagnosis.

The mean AS prevalence per 10 000 (from 36 eligible studies) was 16.7 in Asia^4^. In this part of the world (including Malaysia), a small number of the population tend to obtain treatment from traditional healers for their health problems before eventually seeking conventional professional medical advice. This has resulted in delayed diagnosis and eventual management of patients presenting in advanced stage of diseases, with challenges in treatment and prognosis.

## Case Report

We present a case of a 48-year old man, with undiagnosed ankylosing spondylitis who had been paying frequent visits to a traditional masseuse for his neck pain from which he had been suffering for years. On his most recent visit to the masseuse, the massage produced sharp pain at the nape of his neck and rendered him motionless.

On arrival at the emergency department, the patient was noted to be quadriplegic. At this point of time he was also suffering from bowel and urinary incontinence. Examination of the upper limbs revealed loss of power from C7 with hypoesthesia C5 onwards, atonia and hyporeflexia. There was paralysis of the lower limbs, with atonia, hyporeflexia, hypoesthesia and loss of power from L2 downwards. Anal tone was lax however bulbo-cavernosus reflex (BCR) was present. He was noted to be in neurogenic shock with hypotension requiring ionotropic support.

**Fig. 1:**
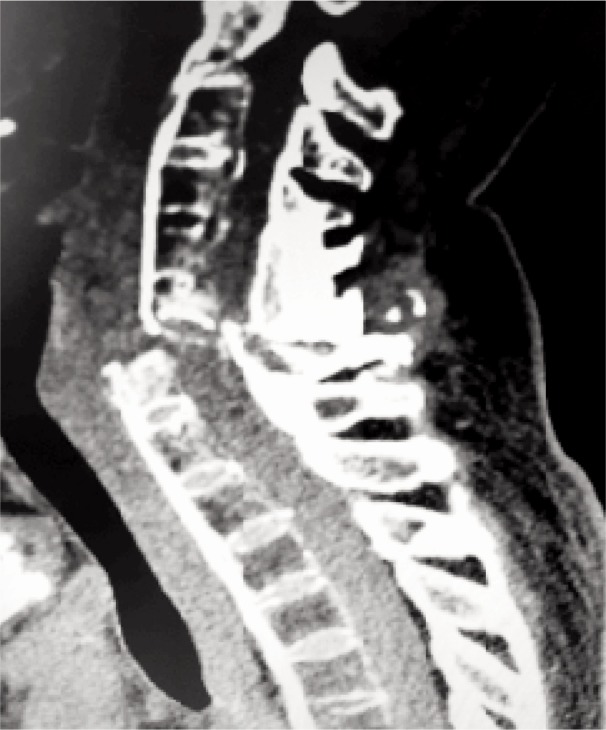
CT cervical spine: sagittal view showing fracture-dislocation of C6 vertebra with facet joint subluxation at C6/C7.

**Fig. 2:**
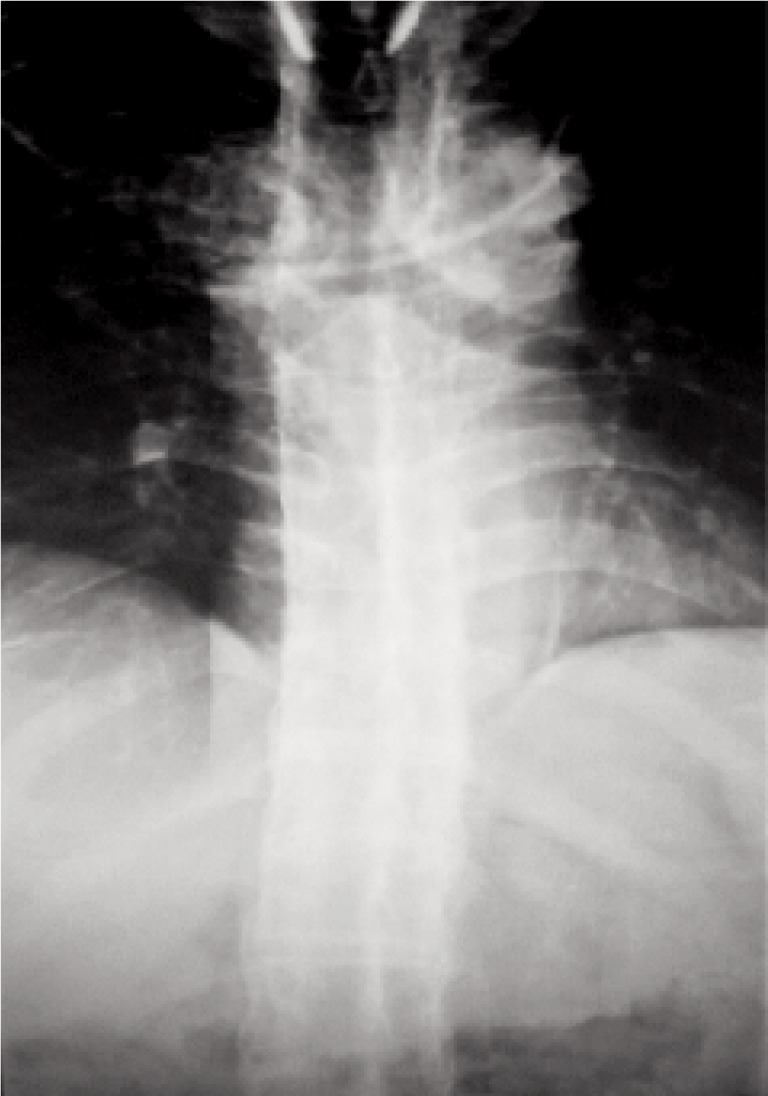
Plain radiograph showing “bamboo spine.”

On suspicion of a high spinal cord injury, an urgent CT of the cervical spine was done (as our facility lacks an MRI machine) (Fig. 1). The scan revealed fracture-dislocation of the C6 vertebra with facet joint subluxation at C6/C7, retrolisthesis, significant canal stenosis, multiple facet and rib joint ankylosis and ligament calcifications with syndesmophytes. Radiographic images of the spine showed the classical “bamboo spine” (Fig. 2) and destruction of the sacroilliac joints bilaterally.

The patient was transferred to a regional spine referral centre where MRI of the cervical spine confirmed the CT findings, with additional information on cord compression causing cord oedema at the level of C6, ligamentous injury, paravertebral and marrow oedema.

**Fig. 3:**
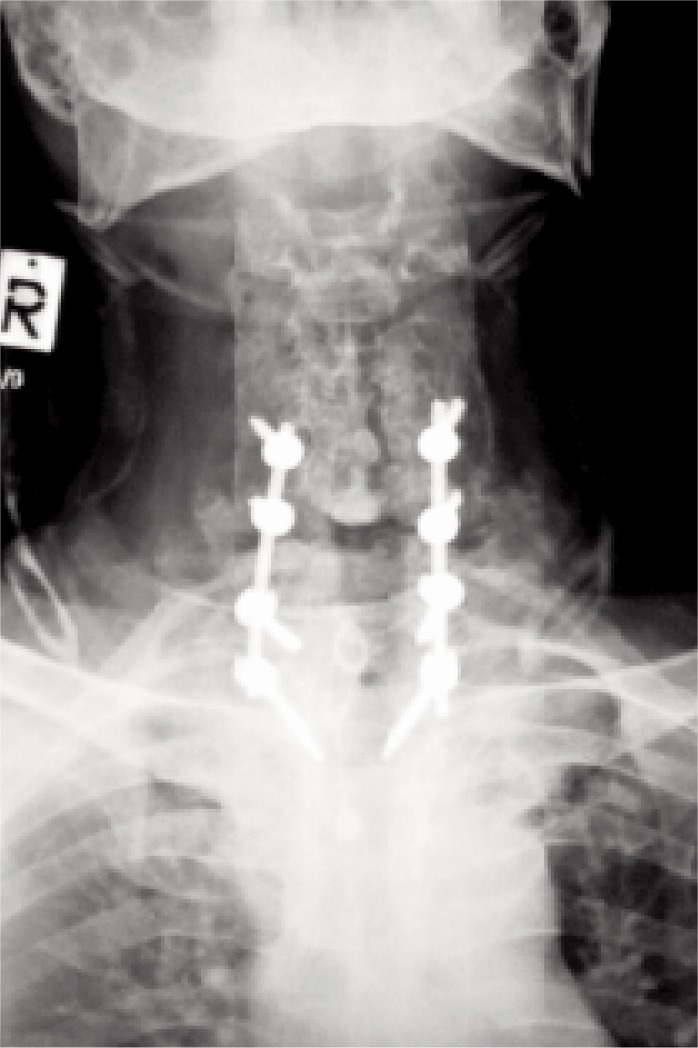
Radiograph following posterior spinal instrumentation and fusion of C4/C5/C7/T1.

He was diagnosed with C6-C7 fracture dislocation with spinal stenosis and retrolisthesis with complete neurology (ASIA A) in neurogenic shock with underlying ankylosing spondylitis. The injury was caused by excessive hyperextension of the neck, which is a common technique practised during the massage. At the spine centre, the patient underwent posterior spinal instrumentation and fusion C4/C5/C7/T1 with laminectomy of C3-C5 (Fig. 3).

Despite our efforts, post-operative assessment showed no change in his neurological state which remained at ASIA A.

The patient is currently undergoing a spinal rehabilitation programme. An interesting finding in this patient was that the HLA-B27 gene tested negative despite fulfilling all points in the Modified New York Criteria for Ankylosing Spondylitis, with radiological appearance of the classical ’bamboo spine” of AS. Statistic show that in the United Kingdom, HLA-B27 is present in 90-95% of patients with ankylosing spondylitis^5^.

## Discussion

Massage therapy is recognized as one of the oldest methods of healing, with references in ancient medical texts going back to 4,000 years. Hippocrates, the father of medicine, had referred to massage when he wrote, in the 4th century B.C.: "The physician must be acquainted with many things, and assuredly with rubbing."

Traditional massage has remained a form of cultural norm especially for a small section of the Asian community for many centuries now. However at times, therapists’ without knowledge on the patients underlying predisposing comorbid conditions and fragilities, tend to push boundaries leading to catastrophe. It was unfortunate that this particular patient with underlying AS went undiagnosed and had resorted to traditional treatment without any knowledge of this condition. A lot of emphasis has been placed on early detection and prevention of such conditions and it is our role to educate and develop awareness in the community to prevent further disasters as in this case.

The majority of cervical spine fractures in ASD are transdiscal extension injuries, most commonly affecting C6–C7 just as observed in our patient. ASD is prone to fracture after minor trauma due to its poor elasticity. Patients with ASD often end up with neurological complications and incomplete neurologic recovery. Most patients with such complications were often unable to cite a specific causative trauma. Timely identification of ankylosing spondylosis is instrumental in avoiding the advanced sequelae of this catastrophic disease.
